# Characteristics of Intermolecular Interactions between Encapsulated Molecules and the Lantern-Like Carcerand Superphanes

**DOI:** 10.3390/molecules29030601

**Published:** 2024-01-26

**Authors:** Mirosław Jabłoński

**Affiliations:** Faculty of Chemistry, Nicolaus Copernicus University in Toruń, Gagarina 7, 87-100 Torun, Poland; teojab@chem.umk.pl; Tel.: +48-056-611-4695

**Keywords:** superphane, cyclophane, carcerand, carceplex, endohedral complex, encapsulation, intermolecular interaction, hydrogen bond, inclusion complex, guest–host interaction

## Abstract

The main topic of the article is to provide the characteristics of individual intermolecular interactions present between three lantern-like superphanes and the $H_{2}$O, ${\mathrm{NH}}_{3}$, HF, HCN, and MeOH molecules trapped inside them. Despite the large cavity, the freedom of the trapped molecules is significantly limited by the presence of numerous interaction sites on the side chains of the superphane molecule. It is shown that the molecule trapped inside the superphane is stabilized mainly by only one or, less often, two strong hydrogen bonds involving the imino nitrogen atom, but QTAIM calculations also suggest the presence of many other intermolecular interactions, mainly hydrogen bonds involving imino or central hydrogen atoms from the side chains of the superphane molecule. Moreover, it is also shown that the structural simplification of the side chains does not significantly affect both the size of the superphane molecule and the obtained encapsulation energies, which is important in modeling this type of carceplexes. Noticeably, the parent superphane considered here was previously synthesized by the group of Qing He, so the results obtained will help in understanding this type and similar systems.

## 1. Introduction

**Figure 1:**
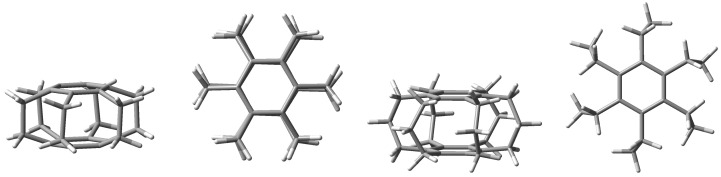
Side and top views of the [2_6_] (**two from the left**) and [3_6_] (**two from the right**) superphanes.

Undoubtedly, superphanes constitute the most unusual subgroup of cyclophanes [1,2,3]. This uniqueness is primarily related to the aesthetically beautiful symmetry of their structure, most often comparable with a six-bladed pinwheel, a lantern, or a barrel. Namely, superphanes consist of two parallel or almost parallel benzene rings (or other aromatic rings [2]) joined together by as many as six bridges [4]. In the simplest representative of superphanes, [2_6_]superphane (i.e., [2_6_](1,2,3,4,5,6)cyclophane), these are ethylene bridges [5,6,7,8,9,10], while the somewhat larger [3_6_]superphane (i.e., [3_6_](1,2,3,4,5,6)cyclophane) has six trimethylene bridges [11,12,13,14,15,16,17,18,19]. The structures of both of these simple superphanes are shown in Figure 1.

**Figure 2:**
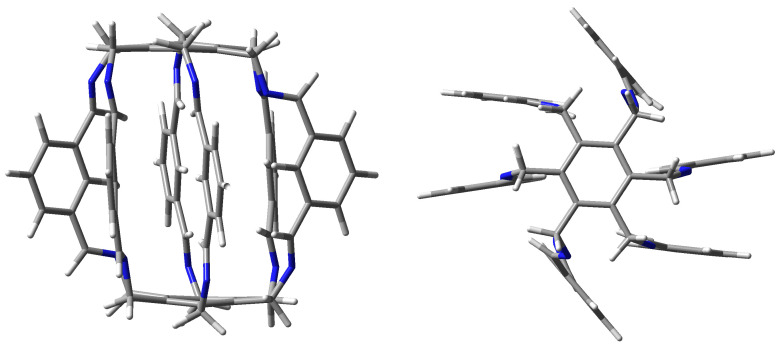
Side and top views of the lantern-like superphane (**1**) synthesized by He et al. [20].

Although the trapping abilities of these superphanes are severely limited or even impossible due to too small size of the internal cavity, one may expect that these abilities increase significantly in larger superphanes with longer side chains [19]. Experimental verification of this possibility may, however, be greatly hampered due to the challenging complexity of the organic synthesis of superphanes. On the other hand, trapping can be greatly facilitated by using multiple binding sites or even charged groups on the side chains of the carcerand superphane molecule. Indeed, very recently, the group of Qing He for the first time synthesized a large-sized lantern-like superphane **1** (Figure 2) and showed that it was able to trap a water dimer [20]. This dimer was stabilized by numerous hydrogen bonds involving disubstituted benzene rings (position 2) and imino protons.

**Figure 3:**
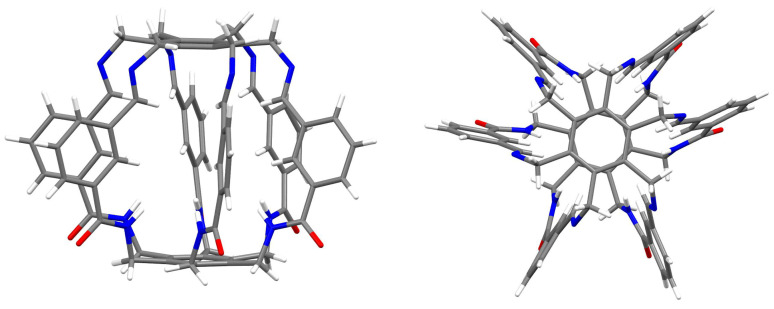
Side and top views of the superphane synthesized by Badjić et al. [25].

Shortly thereafter, the same group in a series of papers showed the possibility of trapping small molecules and anions by various similar superphanes [21,22,23,24]. The trapped species included (2${\mathrm{Cl}}^{-}\cdot$H_2_O) and MeOH [21], ${\mathrm{ReO}}_{4}^{-}$, DMSO, and (H_2_O·MeOH) [22], H_2_${\mathrm{PO}}_{4}^{-}$ and ${\mathrm{AsO}}_{4}^{3-}$ [23], or $I_{2}$ and $I_{3}^{-}$ [24]. The trapped species interact with many binding sites of a given superphane through hydrogen bonds. As has often been emphasized [20,21,22,23,24], the obtained superphanes are characterized by extremely high selectivity towards trapped species over many other types of competing ions, and the obtained carceplexes show high thermal stability in a wide range of pH. Therefore, the obtained superphanes may be extremely important, e.g., in the removal of toxic ions (such as ${\mathrm{AsO}}_{4}^{3-}$) from wastewater. Similar superphanes were also obtained by the group of Jovica D. Badjić [25,26]. Similarly, this group explored the possibility of selectively trapping a wide variety of ions [26], showing that the barrel-shaped hexapodal superphane they obtained (see Figure 3) easily binds tetrahedral oxyanions, such as ${\mathrm{SO}}_{4}^{2-}$ or ${\mathrm{HPO}}_{4}^{-}$ [25]. High selectivity and different ion trapping times result from the specific slotted structure of the side surface of the superphane. It is also noted that the accommodation of the trapped ion is facilitated by the adaptation of the appropriate conformation of the trapping superphane host molecule.

It is also worth mentioning the recent articles by Oh et al. [27] and by Zhao et al. [28]. The obtained carcerands contain only three rather than six side chains, but they also show high recognition towards tetrahedral oxyanions, such as $H_{2}$${\mathrm{PO}}_{4}^{-}$ and ${\mathrm{SO}}_{4}^{2-}$. Thus, these examples are evidence that it is not necessarily the number of side chains closing the internal cavity that is crucial in trapping anions, but rather the number of binding sites. In turn, importantly, these binding sites act actively by forming numerous hydrogen bonds to the trapped species. Thus, these are clear examples of the key role of hydrogen bonds in the recognition and binding of various chemical species.

**Figure 4:**
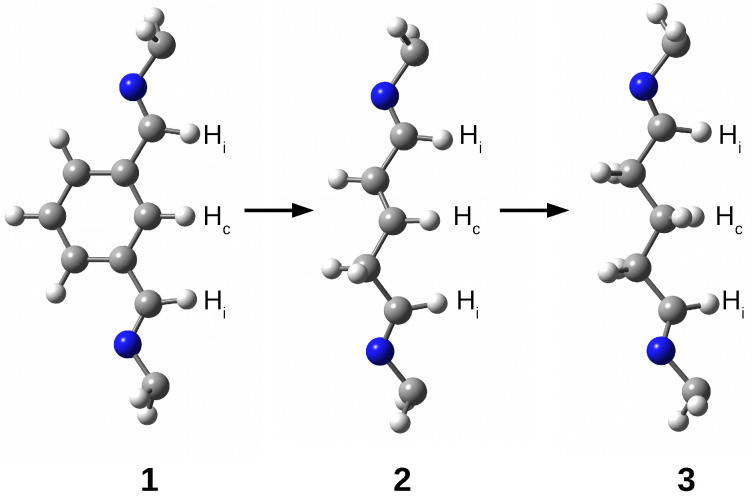
Side chains in superphanes **1**, **2**, and **3**. Hydrogen atoms participating in hydrogen bonds with the guest molecule are labeled as follows: $H_{i}$—imino H atom, $H_{c}$—central H atom.

**Figure 5:**
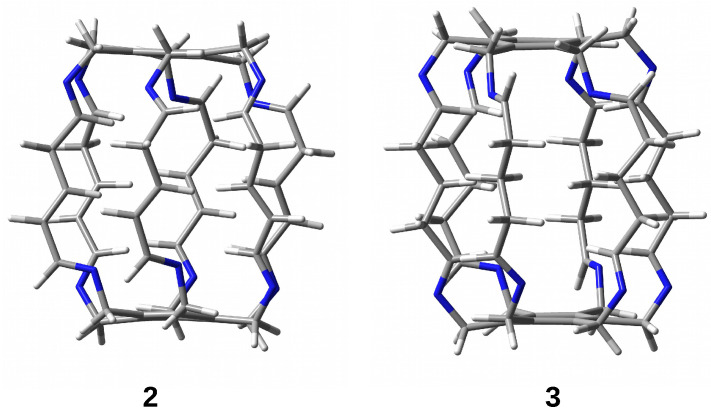
Superphanes **2** and **3**.

The research results show that the trapped species are stabilized inside the superphane cage thanks to many hydrogen bonds [20,21,22,23,24,25,26]. The article aims to characterize the hydrogen bonds formed between five fundamental molecules ($H_{2}$O, ${\mathrm{NH}}_{3}$, HF, HCN, and MeOH) trapped in the cavity of superphane **1** and the donor and acceptor centers of this superphane molecule. In addition to the geometric parameters of the found hydrogen bonds, an important issue will be the total guest⋯host interaction energy, and especially the estimated interaction energies of the most important individual hydrogen bonds and other intermolecular interactions found. In contrast to the unavailability of this parameter experimentally, the energy of an individual interaction can be relatively easily estimated based on theoretical studies. Another important goal is to investigate the influence of the type of side chain on the structure of the obtained carceplexes, especially on the pattern of the hydrogen bonds formed and their energy. For this reason, the side chains in the parent superphane **1** were gradually reduced to those present in superphanes **2** and **3**. The **1** → **2** step involves replacing the benzene ring in the side chains with the -CH=CH-${\mathrm{CH}}_{2}$- fragment having a double bond, while the **2** → **3** replacement involves saturating this bond, i.e., introducing the -C$H_{2}$-C$H_{2}$-C$H_{2}$- fragment (see Figure 4). As a result of these changes, the obtained superphanes **2** and **3** have a significantly simplified structure with the lack of as many as six side benzene rings (Figure 5), but the binding sites are retained (Figure 4).

## 2. Results and Discussion

### 2.1. Characteristics of Intermolecular Interactions in Carceplexes Guest@***1***

**Figure 6:**
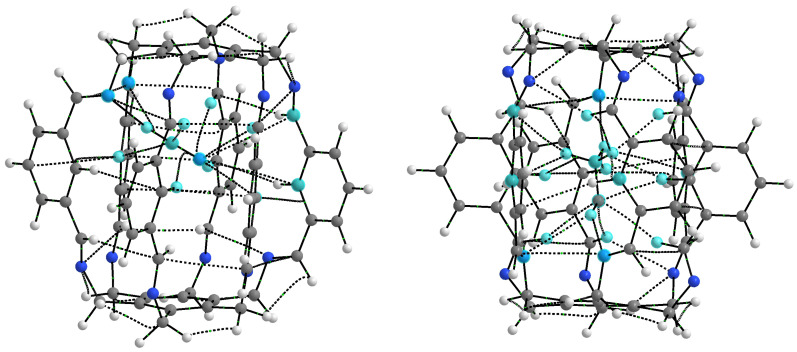
Molecular graphs (ring critical points and cage critical points have been removed for better clarity) of carceplexes **14a** (i.e., the form **a** of HCN@**1**) and **15a** (i.e., the form **a** of MeOH@**1**). Atoms of the guest molecule and all host atoms connected to them through bond paths are marked in light blue.

As seen from Figure 2 and Figure 5, the considered superphanes **1**, **2**, and **3** are large enough that the guest molecules ($H_{2}$O, ${\mathrm{NH}}_{3}$, HF, HCN, or MeOH) trapped inside them have quite a lot of freedom. However, this freedom should be somewhat suppressed by the presence of distinct binding sites, such as the imino nitrogen and the proton from either the central or the imino C-H bond (Figure 4). The specific slotted structure of superphanes and their limited internal spaces result in relatively small distances between the atoms of the superphane and the guest molecule. Consequently, the molecular graphs obtained for the considered carceplexes quite often feature numerous bond paths between the superphane atoms and the molecules trapped in them, thus suggesting the occurrence of many intermolecular interactions. The multitude and diversity of intermolecular interactions are visible in the representative molecular graphs of **14a** and **15a**, i.e., the most stable forms of the carceplexes HCN@**1** and MeOH@**1**, respectively (Figure 6). The former of these systems contains 11 bond paths between the trapped guest molecule (i.e., HCN) and the superphane, while the latter one features as many as 17 such bond paths.

The general characteristics (type, values of the electron density at the bond critical point ($\rho_{\mathrm{bcp}}$), interaction energies according to Equation (2) (BE), and their sum (∑BE)) of the various individual interactions between the guest molecule and the superphane atoms indicated by the presence of corresponding bond paths are given in Table 1. Additionally, the encapsulation energy ($E_{b}$) (Equation (1)) is also shown.

#### 2.1.1. Intermolecular Interactions in $H_{2}$O@**1**

**Figure 7:**
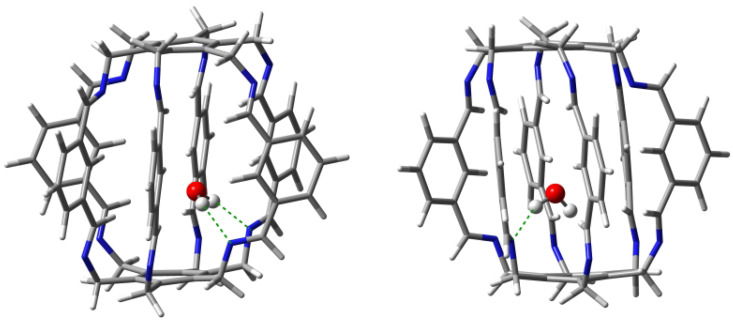
Structures of carceplexes **11a** (**left**) and **11b** (**right**). The dominant N⋯H-O hydrogen bonds are marked with a green dashed line.

Geometry optimizations of several starting $H_{2}$O@**1** structures led to two forms, **11a** and **11b** (Figure 7), with clearly different characteristics of the intermolecular interactions between the trapped water molecule and the superphane **1** (Table 1). The binding energy of the more stable form **11a** (−18.8 kcal/mol) is dominated by two strong (−5.9 and −4.7 kcal/mol) hydrogen bonds between the O-H bonds of the water molecule and the nitrogen atoms of the imino groups of the superphane **1**. The N⋯H distances are 1.969 and 2.068 Å, respectively, and the N-H-O angles are $164^{\circ }$ and $148^{\circ }$. So both of these hydrogen bonds are far from linear (Figure 7). According to QTAIM (i.e., Quantum Theory of Atoms in Molecules [29,30,31]), the water molecule in **11a** is additionally weakly (BE is from −0.4 to −1.4 kcal/mol) stabilized by as many as four hydrogen bonds between the oxygen atom of water and the C-H bonds of **1**, with one hydrogen atom coming from the imino group, while as many as three from the benzene rings in the side chains. The total binding energy of all the interactions demonstrated by the presence of bond paths is −14.2 kcal/mol.

The slightly less stable ($E_{b}$ = −16.8 kcal/mol) form **11b** is dominated by only one N⋯H-O hydrogen bond with an energy of −4.1 kcal/mol, while the second N⋯H-O hydrogen bond is, according to QTAIM, very weak (only −0.6 kcal/mol). This is also visible in the values of the lengths and angles of both hydrogen bonds, which are 2.108 and 2.724 Å and $154^{\circ }$ and $120^{\circ }$, respectively. Additionally, according to QTAIM, the water molecule is also stabilized by two C-$H_{i}\cdots$O hydrogen bonds and two C-$H_{c}\cdots$H-O interactions, for which BE is from −1.0 to −2.2 kcal/mol (Table 1). Comparison of the type of bond paths in carceplexes **11a** and **11b** shows that changing the position of the water molecule causes the C-$H_{c}\cdots$O bond paths to ’switch’ to C-$H_{c}\cdots$H-O. Moreover, the molecular graph for **11b** also shows energetically irrelevant C(π)⋯O and C-$H_{i}\cdots$H-O bond paths. The total binding energy of all the intermolecular interactions traced by bond paths is −11.6 kcal/mol.

Taking into account the results obtained for both forms of carceplex $H_{2}$O@**1**, it can be concluded that the trapped water molecule is stabilized mainly by two (in **11a**) or one (in **11b**) strong N⋯H-O hydrogen bonds, but these bonds are accompanied by numerous interactions that involve imino or central C-H bonds.

#### 2.1.2. Intermolecular Interactions in ${\mathrm{NH}}_{3}$@**1**

**Figure 8:**
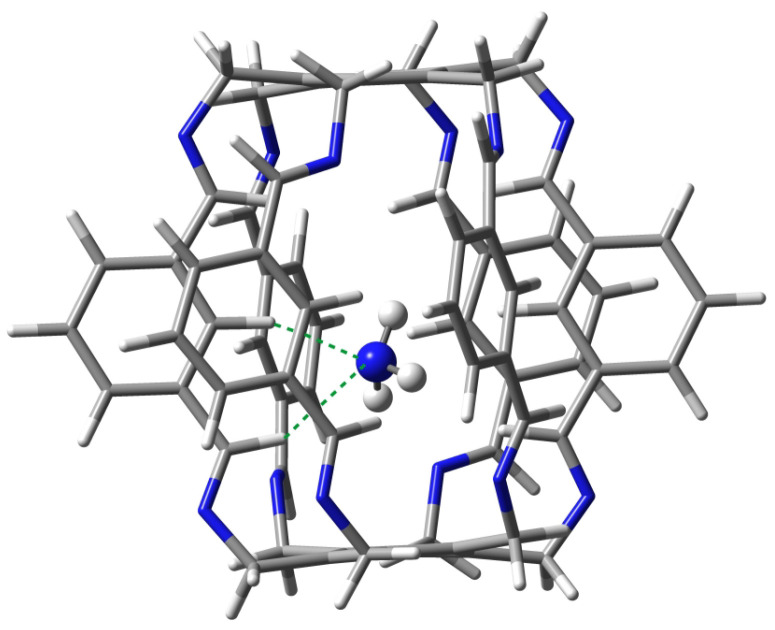
Structure of the **12** (i.e., N$H_{3}$@**1**) carceplex. The green dashed lines indicate the two strongest C-H⋯N hydrogen bonds, with BEs of −3.4 and −1.8 kcal/mol.

Carceplex ${\mathrm{NH}}_{3}$@**1** is the only one for which only one form was obtained (Figure 8). The interaction between ${\mathrm{NH}}_{3}$ and **1** is weaker (−12.9 kcal/mol) than the previously discussed $H_{2}$O⋯1 in $H_{2}$O@**1**. The ${\mathrm{NH}}_{3}\cdots 1$ interaction is mainly dominated by C-$H_{c}\cdots$N hydrogen bonds (BE of the strongest of them is −3.4 kcal/mol) and, to a much lesser extent, C-$H_{i}\cdots$N (−1.8 kcal/mol). According to QTAIM, the trapped ammonia molecule interacts with the superphane **1** also through its N-H bonds, forming two N⋯H-N hydrogen bonds (BE are −0.6 and −1.4 kcal/mol) and two weak C-$H_{i}\cdots$H-N contacts (−0.2 and −1.1 kcal/mol). Despite the relative weakness of the interactions (perhaps except one C-$H_{c}\cdots$N hydrogen bond), their large number (10) makes the ∑BE value relatively large at −9.9 kcal/mol.

Both C-H⋯N hydrogen bonds marked with the green dashed line in Figure 8 are quite long, 2.272 and 2.489 Å. These bonds are also clearly far from linear, the C-H-N angle being $158^{\circ }$ and $152^{\circ }$, respectively.

#### 2.1.3. Intermolecular Interactions in HF@**1**

**Figure 9:**
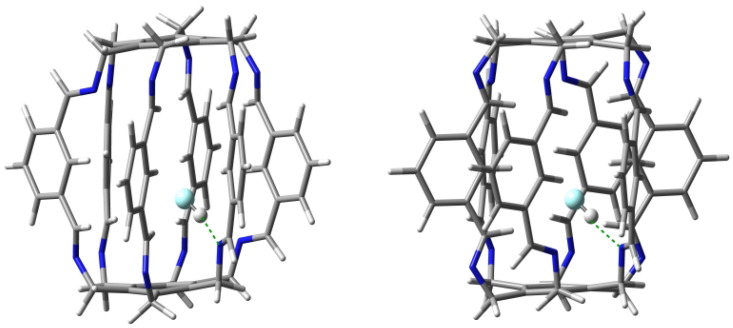
Structures of carceplexes **13a** (**left**) and **13b** (**right**). The dominant N⋯H-F hydrogen bonds are marked with a green dashed line.

Carceplexes HF@**1** deserve special attention. Although the total binding energy (−19.7 kcal/mol for form **13a** and −18.5 kcal/mol for form **13b**) is only slightly higher than for the $H_{2}$O@**1** carceplexes, the HF⋯1 binding is mainly due to the formation of the very strong (−10.8 kcal/mol in **13a** and −9.5 kcal/mol in **13b**) hydrogen bond N⋯H-F. This is also visible in the very short N⋯H distance, which is 1.701 and 1.747 Å in **13a** and **13b**, respectively. This is a clear confirmation of the fact that the HF molecule is an excellent proton donor. This has also been recently demonstrated with examples of hydrogen bonds to the carbene electron lone pair [32]. The N⋯H-F hydrogen bonds in **13a** and **13b** are closer to being linear, the N-H-F angle amounts to $175^{\circ }$ and $166^{\circ }$, respectively. The structures of both obtained forms of carceplex HF@**1** (i.e., **13a** and **13b**) along with N⋯H-F hydrogen bonds are shown in Figure 9.

The HF molecule contains a strongly electronegative fluorine atom with electron lone pairs. It is therefore not surprising that the obtained molecular graphs suggest the presence of many hydrogen bonds to this atom (see Table 1). Namely, in the case of both forms, the molecular graphs show two C-$H_{i}\cdots$F bond paths and three C-$H_{c}\cdots$F bond paths. Although, of course, these hydrogen bonds are much weaker (up to −2.0 kcal/mol in **13b**) than N⋯H-F, their multitude suggests a not necessarily negligible contribution to the total binding of the trapped HF molecule. The ∑BE values are −16.4 and −14.7 kcal/mol in **13a** and **13b**, respectively.

#### 2.1.4. Intermolecular Interactions in HCN@**1**

**Figure 10:**
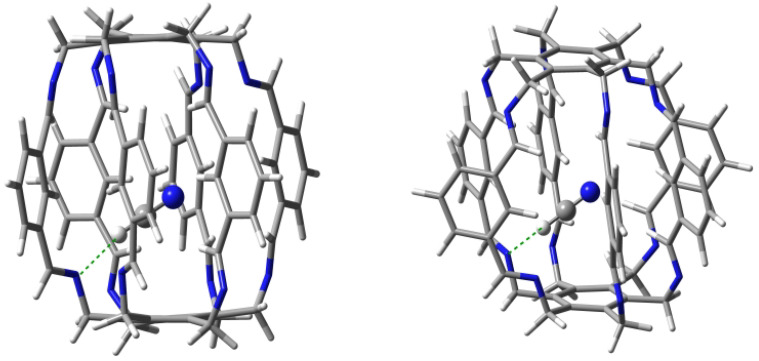
Structures of carceplexes **14a** (**left**) and **14b** (**right**). The dominant N⋯H-C hydrogen bonds are marked with a green dashed line.

The HCN molecule can be both a proton donor and a proton acceptor, so it was interesting to see its main sites of interaction after trapping. Geometry optimizations led to two forms of the HCN@**1** carceplex with equal binding energies (−16.2 kcal/mol) and very similar arrangements of the HCN molecule (Figure 10).

QTAIM calculations indicate a clear dominance of one of the two N⋯H-C hydrogen bonds (in **14a**, one of the bond paths leads to the critical point of the H-C bond of the HCN molecule). Its energy is −5.3 and −5.8 kcal/mol in **14a** and **14b**, respectively. Its length is 2.007 and 1.984 Å, respectively, and the N-H-C angle is $166^{\circ }$ and $155^{\circ }$, respectively, so the slightly shorter and stronger bond in **14b** is more bent. The second N⋯H-C hydrogen bond is much weaker, with an energy of only ca. −1 kcal/mol. However, this leading N⋯H-C type hydrogen bond is accompanied by many different interactions with the C atom, and especially N (Table 1), which results, of course, from the presence of an electron lone pair on this atom. This situation is well illustrated by the molecular graph of carceplex **14a** shown earlier in Figure 6. Among these interactions, the greatest stabilization comes from one C-$H_{c}\cdots$N hydrogen bond (−2.6 and −2.3 kcal/mol in **14a** and **14b**, respectively), again showing that the central C-H bond (Figure 4) is a good proton donor. The ∑BE values are ca. −14 kcal/mol and are only slightly lower than the $E_{b}$ value (−16.2 kcal/mol).

#### 2.1.5. Intermolecular Interactions in MeOH@**1**

The MeOH molecule is the largest of all guest molecules considered here. Additionally, it contains an extensive methyl group, so the presence of many different bond paths between this molecule and the host superphane **1** should be expected. Indeed, this is confirmed by the results presented in Table 1 and the molecular graph shown for **15a** in Figure 6. This form features as many as four different interactions stronger than −1.5 kcal/mol. These are: N⋯H-O (−1.9 kcal/mol), C-$H_{c}\cdots$H-C (−1.8 kcal/mol), C-$H_{c}\cdots$O (−1.7 kcal/mol), and C-$H_{c}\cdots$(H-C) (−1.6 kcal/mol). However, the molecular graph (Figure 6) also shows the presence of three bond paths for the C-$H_{i}\cdots$O interaction, three for C(π)⋯H-C, three for C-$H_{i}\cdots$H-C, and one for $C_{i}\cdots$H-C and N⋯H-C. Although these interactions are much weaker (and some are even slightly destabilizing according to Emamian’s formula 2), the energy of some of them is ca. −1 kcal/mol. The binding energy of all interactions traced by the presence of bond paths is −13.0 kcal/mol, much lower (by ca. 6 kcal/mol) than the encapsulation energy (−19.2 kcal/mol), showing that other long-range interactions are also important.

The molecular graph of the second found form of MeOH@**1** carceplex, i.e., **15b**, is considerably poorer in the variety of bond path types (Table 1). Somewhat surprisingly, according to QTAIM, the strongest are two C-$H_{c}\cdots$O hydrogen bonds with energies of −2.1 and −2.0 kcal/mol (the third C-$H_{c}\cdots$O is weaker with BE of −1.4 kcal/mol), while the standard N⋯H-O hydrogen bonds have energies of only −1.5 and −0.6 kcal/mol. Interactions with the methyl group in MeOH are also relatively important, especially with the participation of (two) protons of the imino groups. The binding energies of both these C-$H_{i}\cdots$H-C contacts are −1.5 kcal/mol. Although the total number of interactions indicated by the presence of bond paths is much smaller (12) than in **15a** (17), the ∑BE value is similar (−13.3 kcal/mol).

**Figure 11:**
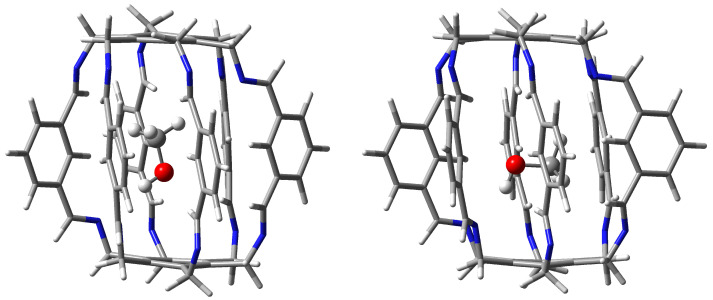
Structures of carceplexes **15a** (**left**) and **15b** (**right**).

A large number of diverse (weak) interactions results in the fact that the encapsulation energies of these carceplexes (−19.2 and −18.3 kcal/mol for **15a** and **15b**, respectively) belong to the highest among all obtained and are similar to those for HF@**1**. It is quite significant that the standard N⋯H-O hydrogen bond, i.e., to the highly polarized O-H bond, is, according to QTAIM, not the dominant interaction (especially in **15b**). Instead, QTAIM calculations suggest a large energetic contribution from interactions involving the oxygen atom and the methyl group of the MeOH molecule. It is worth adding that the distances between the hydroxyl hydrogen atom of methanol and the nearest nitrogen atoms in the superphane are quite large; in the case of carceplex **15a**, the distances are 2.400 and as much as 2.844 Å, while in the case of **15b**, 2.468 and as much as 2.702 Å. Moreover, the N⋯H-O hydrogen bonds are significantly bent. It can therefore be assumed that the large nonlinearity of the N⋯H-O hydrogen bonds together with their considerable length is the reason for their weakness. The structures of the obtained MeOH@**1** carceplex forms (i.e., **15a** and **15b**) are shown in Figure 11.

### 2.2. Influence of the Type of Side Chain

Another aim of the research on the carceplexes considered here was to investigate the influence of the type of side chain on the height of the superphane molecule and the strength of the guest⋯superphane interaction. These issues will be discussed in the next two subsections.

#### 2.2.1. Influence of the Type of Side Chain on the Height of the Cage

Table 2 shows the values of the distance between the centers of opposite benzene rings, which can be taken as a parameter describing the height of the cage created by the superphane molecule.

It is worth noting first that the structural reduction of side chains in the superphane molecule (see Figure 4) leads to a slight decrease in the cage’s height, most significantly in the case of **1** → **2** (from 9.15 to 8.96 Å). Encapsulation does not cause significant changes in the height of the superphane molecule cage. This result confirms the relatively large cavity space and stiffness of the side chains.

#### 2.2.2. Influence of the Type of Side Chain on the Encapsulation Energy

The obtained encapsulation energy values of all considered carceplexes are given in Table 3.

The obtained results show that the structural reduction of the side chains of superphane **1** does not significantly affect the encapsulation energies. This result is important as it shows that the guest@**1** carceplexes can be successfully modeled by the less computationally demanding guest@**2** and guest@**3** carceplexes. Importantly, except for MeOH, the **1** → **2** exchange leads to an increase in the encapsulation energies (i.e., they become more negative) of the most stable forms, while the **1** → **3** exchange leads to a decrease in these energies (i.e., they become less negative). Thus, the guest molecule is most stable when trapped by superphane **2** and least stable in a carceplex formed by superphane **3**. It would be interesting to see whether this relationship would hold for the species considered by Qing He’s [20,21,22,23,24] and Jovica D. Badjić’s [25,26] groups, but so far superphanes **2** and **3** have not been synthesized yet.

**Figure 12:**
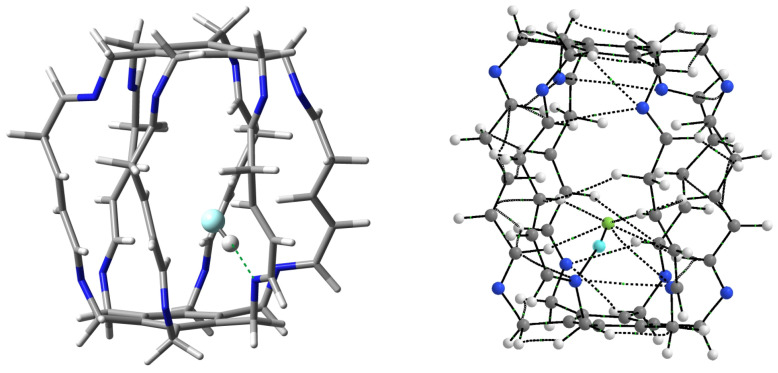
Structure (**left**) and molecular graph (**right**) of the most stable HF@**2** (i.e., **23**) carceplex. In the subfigure on the left, the most important N⋯H-F hydrogen bond is marked with a green dashed line. In turn, in the molecular graph on the right, ring critical points and cage critical points have been removed for greater clarity. Additionally, the hydrogen atom of the HF molecule is highlighted in light blue.

The greatest increase in stabilization occurred for the HF molecule (from −19.7 kcal/mol in **13a** to −23.1 kcal/mol in **23**, i.e., by 3.4 kcal/mol), so it is particularly worth taking a closer look at this case. The structure and molecular graph of the most stable carceplex **23** are shown in Figure 12.

Similarly to carceplexes **13a** and **13b**, the stabilization of the system is dominated by the N⋯H-F hydrogen bond, but in the case of carceplex **23**, this bond is much stronger. According to Equation (2), its energy is −12.1 kcal/mol, while −10.8 and −9.5 kcal/mol in **13a** and **13b**, respectively. The exceptional strength of this bond is also reflected in its extremely short length, which is only 1.657 Å. QTAIM calculations suggest, however, that such a high stabilization of carceplex **23** is also due to the significant participation of three relatively strong C-H⋯F hydrogen bonds, two involving the central H atoms (−2.7 and −2.0 kcal/mol) and one involving hydrogen from the imino group (−1.6 kcal/mol). The remaining interactions visible in Figure 12 are not significant (−0.8 kcal/mol for the third hydrogen bond C-$H_{c}\cdots$F, −0.2 kcal/mol for the second hydrogen bond C-$H_{i}\cdots$F, and −0.6 kcal/mol for the contact N⋯F). The total binding energy for all HF⋯**2** interactions visible in Figure 12 is −20.1 kcal/mol, which is greater than −16.4 and −14.7 kcal/mol for **13a** and **13b**, respectively (Table 1).

On the contrary, the strongest weakening via the **1** → **3** structural modification has occurred for water. Namely, the encapsulation energy decreased from −18.8 kcal/mol in **11a** to −16.6 kcal/mol in **31a**, i.e., by 2.2 kcal/mol. QTAIM calculations suggest that this weakening of carceplex stabilization is mainly due to a slight weakening of both N⋯H-O hydrogen bonds, from −5.9 and −4.7 kcal/mol in **11a** to −5.1 and −4.3 kcal/mol in **31a**, however, this weakening is partially compensated by slightly stronger C-H⋯O bonds (their total binding energy is −5.3 kcal/mol in **31a** while only −3.6 kcal/mol in **11a**). The weakening of the N⋯H-O bonds is also suggested by their lengthening, from 1.969 and 2.068 Å in **11a** to 2.033 and 2.094 Å in carceplex **31a**. Moreover, both of these bonds are strongly non-linear ($158^{\circ }$ and $154^{\circ }$, respectively).

## 3. Methodology

Geometry optimizations of the superphanes **1** (see Figure 2), **2** and **3** (see Figure 5) and their carceplexes with $H_{2}$O, N$H_{3}$, HF, HCN, or MeOH were performed at the ωB97X-D/6-31G(d) level of theory, i.e., within the ωB97X-D exchange-correlation functional [33] of Density Functional Theory [34,35] and the 6-31G(d) basis set [36,37]. As shown [38], within the group of the 200 tested exchange-correlation functionals, ωB97X-D proved to be one of the best for general purposes, including the reliable description of intermolecular interactions. Moreover, as shown earlier [9], this functional well reproduced the crystallographic structure of [2_6_]superphane. Frequency analysis were performed to confirm true minima on the potential energy surface. In each case, all frequencies were positive, confirming that stable forms were always obtained. It should be noted that the multiplicity of binding sites in the side chains of the tested superphanes may lead to many minima on the potential energy surface, i.e., to various forms of the obtained carceplexes. Therefore, geometry optimizations have started with several different initial geometries, which in some cases have led to different final carceplex geometries, although in some cases only one such complex has been obtained. For convenience, the obtained guest@superphane carceplexes have been marked as **ijk**, where **i** denotes the superphane molecule (i.e., **1**, **2**, or **3**; see Figure 2 and Figure 5), **j** numbers the guest molecule, **1** for $H_{2}$O, **2** for N$H_{3}$, **3** for HF, **4** for HCN, and **5** for MeOH, while **k**, being **a**, **b**, or possibly **c**, refers to the corresponding forms of carceplex **ij** in order from the most stable to the least stable.

Despite the relatively small size of the basis set used for geometry optimization (due to the large size of the considered superphanes, many degrees of freedom, and therefore high computational cost), the total energies were then calculated (single point calculations) using larger 6-31++G(d) basis set, containing diffuse functions on all atoms. Geometry optimizations, frequency analysis, and single point calculations were performed using the Gaussian 16 package [39]. Graphical representations of the systems were obtained with the GaussView 6 program [40].

The encapsulation energy was calculated as the difference between the total energy of the complex (carceplex) and the sum of the total energies of the superphane and the guest molecule trapped in it:

(1) $$E_{b}=E\left(\mathrm{carceplex}\right)-E\left(\mathrm{superphane}\right)-E\left(\mathrm{guest}\right)$$

One of the goals of this work is to assess the strength of individual hydrogen bonds and other intermolecular interactions involved in the overall superphane⋯guest binding. Determining the energies of individual hydrogen bonds is not easy using experimental methods, but it is relatively simple using tools of theoretical chemistry. For this purpose, Espinosa’s formula $E=\frac{1}{2}V_{\mathrm{bcp}}$ [41] is most often used, based on the value of the potential energy density determined at the so-called bond critical point (bcp) [29,30,31] of a given bond (interaction). However, due to numerous objections to this formula (see [42] and especially point 2.5.1 in the review article [43]), it was not used here. Firstly, due to the negativity of $V_{\mathrm{bcp}}$, the obtained value of *E* is always negative, thus describing each interaction as stabilizing. Secondly, it should be recalled that Espinosa’s formula was actually derived only for X-H⋯O hydrogen bonds (X = C, N, O) and its application to other types of interactions is not necessarily correct. For this reason, improved versions of this formula can be found in the literature [42,44,45]. It can also be objected that the values of fitting bond energies were obtained using a mixture of various theoretical methods. On the other hand, Emamian et al. [46] showed that among various wave function–based descriptors of hydrogen bonds, the electron density determined at the bond critical point correlates best with the binding energy. Therefore, their Equation (2) was used in this work:

(2) $$\mathrm{BE}=-223.08\cdot \rho_{\mathrm{bcp}}+0.7423$$

where $\rho_{\mathrm{bcp}}$ is the value (in au) of the electron density at bcp of a given hydrogen bond. As Emamian et al. emphasized in the abstract of their article [46], this formula can be used to estimate the binding energy of individual hydrogen bonds, e.g., in biomolecules. It should be noted, however, that, as Equation (2) shows, the value of the electron density at the bond critical point itself could in principle be used as a measure of the strength of a given interaction. Molecular graphs and electron density values at the bond critical points of individual interactions were obtained using the AIMAll program [47].

## 4. Conclusions

The main topic of the article was to provide the characteristics of intermolecular interactions present between trapped $H_{2}$O, N$H_{3}$, HF, HCN, or MeOH molecules and the lantern-like superphane (**1**) acting as a host, previously synthesized by the group of Qing He [20]. It has been shown that despite the large internal cavity of the superphane molecule, the freedom of the trapped molecules is significantly limited by the presence of numerous interaction sites. In addition to imino nitrogen atoms, which most often form only one or, rarely, two strong hydrogen bonds to the trapped guest molecule, the presence of numerous weaker hydrogen bonds involving central or imino C-H bonds deserves special attention. QTAIM calculations suggest that other interactions indicated by the presence of bond paths are less important.

It has also been shown that a slight structural simplification of the side chains of the encapsulating superphane **1** (consequently leading to superphanes **2** and then **3**) has no significant effect on the cage height and has only a minor effect on the encapsulation energy of a given guest molecule. Therefore, it is concluded that the carceplexes obtained by the Qing He group as well as other similar systems can be successfully modeled by using these slightly simplified superphanes **2** and **3**. The use of these superphanes also leads to a lower computational cost while, as shown, maintaining the main conclusions regarding the characteristics of the interactions in the guest@**1** system.

## Figures and Tables

**Table 1:** Characteristics of individual intermolecular interactions in carceplexes guest@**1**. The dominant interactions and their energies (BE) are marked in boldface.

Carceplex	SP	Guest	$E_{b}$ ^a^	∑BE ^b^	Intermolecular Interaction			
					*n* ^c^	Type ^d^	$\rho_{\mathrm{bcp}}$ ^e^	BE ^f^
**11a**	**1**	$H_{2}$O	−18.8	−14.2	2	**N⋯H-O**	0.024 and 0.030	**−4.7** and **−5.9**
					1	C-$H_{i}\cdots$O	0.006	−0.7
					3	C-$H_{c}\cdots$O	0.005–0.010	−0.4 to −1.4
**11b**	**1**	$H_{2}$O	−16.8	−11.6	2	**N⋯H-O**	0.006 and 0.022	−0.6 and **−4.1**
					2	C-$H_{i}\cdots$O	0.010 and 0.013	−1.5 and −2.2
					2	C-$H_{c}\cdots$H-O	0.008 and 0.010	−1.0 and −1.6
					1	C(π)⋯O	0.006	−0.6
					1	C-$H_{i}\cdots$H-O	0.004	0.0
**12**	**1**	N$H_{3}$	−12.9	−9.9	1	C-$H_{i}\cdots$N	0.012	−1.8
					5	**C-H_c_⋯N**	0.002–0.019	0.3 to **−3.4**
					2	C-$H_{i}\cdots$H-N	0.004 and 0.008	−0.2 and −1.1
					2	N⋯H-N	0.006 and 0.010	−0.6 and −1.4
**13a**	**1**	HF	−19.7	−16.4	1	**N⋯H-F**	0.052	**−10.8**
					2	C-$H_{i}\cdots$F	0.006 and 0.010	−0.5 and −1.5
					3	C-$H_{c}\cdots$F	0.004–0.010	−0.2 to −1.6
					1	C(π)⋯F	0.003	0.2
					1	N⋯F	0.006	−0.7
**13b**	**1**	HF	−18.5	−14.7	1	**N⋯H-F**	0.046	**−9.5**
					2	C-$H_{i}\cdots$F	0.004 and 0.012	−0.2 and −2.0
					3	C-$H_{c}\cdots$F	0.003–0.011	0.1 to −1.7
					1	N⋯F	0.007	−0.8
**14a**	**1**	HCN	−16.2	−13.9	1	**N⋯H-C**	0.027	**−5.3**
					1	N⋯(H-C)	0.009	−1.2
					1	C-$H_{c}\cdots$C	0.005	−0.4
					1	C-$H_{i}\cdots$C	0.010	−1.5
					3	C-$H_{c}\cdots$N	0.004–0.015	−0.2 to −2.6
					2	C-$H_{i}\cdots$N	0.006 and 0.008	−0.5 and −1.2
					1	C(π)⋯N	0.003	0.1
					1	$C_{i}\cdots$N	0.002	0.2
**14b**	**1**	HCN	−16.2	−14.6	2	**N⋯H-C**	0.008 and 0.029	−1.1 and **−5.8**
					1	C-$H_{c}\cdots$C	0.007	−0.8
					1	C-$H_{i}\cdots$C	0.009	−1.2
					1	C(π)⋯C	0.004	0.0
					3	C-$H_{c}\cdots$N	0.005–0.014	−0.4 to −2.3
					2	C-$H_{i}\cdots$N	0.006–0.008	−0.6 and −0.9
					1	C(π)⋯N	0.002	0.3
**15a**	**1**	MeOH	−19.2	−13.0	2	**N⋯H-O**	0.004 and 0.012	−0.2 and **−1.9**
					1	**C-H_c_⋯O**	0.011	**−1.7**
					3	C-$H_{i}\cdots$O	0.003–0.008	0.2 to −1.1
					3	C(π)⋯H-C	0.001–0.009	0.5 to −1.2
					2	**C-H_c_⋯H-C**	0.002 and 0.011	0.3 and **−1.8**
					3	C-$H_{i}\cdots$H-C	0.007–0.009	−0.8 to −1.2
					1	**C-H_c_⋯(H-C)**	0.010	**−1.6**
					1	$C_{i}\cdots$H-C	0.005	−0.5
					1	N⋯H-C	0.003	0.1
**15b**	**1**	MeOH	−18.3	−13.3	2	N⋯H-O	0.006 and 0.010	−0.6 and −1.5
					3	**C-H_c_⋯O**	0.010–0.013	−1.4 to **−2.1**
					1	C(π)⋯O	0.004	−0.1
					2	C-$H_{i}\cdots$H-C	0.010 and 0.010	−1.5 and −1.5
					2	$C_{i}\cdots$H-C	0.007 and 0.008	−0.8 and −1.0
					2	C(π)⋯H-C	0.005 and 0.006	−0.3 and −0.7

^a^ Encapsulation energy (in kcal/mol) given by Equation (1). ^b^ Total binding energy (in kcal/mol) obtained by summing over individual interactions, i.e., ∑BE = ∑_*i*_BE_*i*_. ^c^ Number of intermolecular interactions of a given type. ^d^ Type of a given interaction in the format atom/bond(superphane)⋯atom/bond(guest). The subscripts i and c refer to the imino group and the central position, respectively (see Figure 4), while C(*π*) denotes the carbon atom from the central benzene ring. ^e^ The value of the electron density (in au) at the bond critical point (bcp) of a given interaction. ^f^ Binding energy (in kcal/mol) according to Equation (2) for a single interaction.

**Table 2:** The distance (in Å) between the centers of the two confining benzene rings of a superphane.

Superphane		H_2_O			NH_3_		HF		HCN		MeOH	
		a	b	c	a	b	a	b	a	b	a	b
**1**	9.15	9.18	9.16	n/a	9.11	n/a	9.21	9.18	9.10	9.10	9.08	9.05
**2**	8.96	8.97	9.01	n/a	8.91	8.93	9.03	n/a	8.94	n/a	8.81	n/a
**3**	9.05	9.09	9.07	9.08	9.02	9.04	9.12	9.10	9.02	n/a	8.99	n/a

**Table 3:** Encapsulation energies (in kcal/mol) for various forms of the guest@superphane carceplexes.

Superphane	H_2_O			NH_3_		HF		HCN		MeOH	
	a	b	c	a	b	a	b	a	b	a	b
**1**	−18.8	−16.8	n/a	−12.9	n/a	−19.7	−18.5	−16.2	−16.2	−19.2	−18.3
**2**	−19.6	−16.5	n/a	−13.1	−13.0	−23.1	n/a	−18.4	n/a	−17.5	n/a
**3**	−16.6	−15.6	−14.2	−12.9	−12.5	−18.3	−16.4	−15.8	n/a	−18.0	n/a

## Data Availability

Data available from the author on reasonable request.
